# Simultaneous and cumulative effects of tDCS on cerebral metabolic rate of oxygen in multiple sclerosis

**DOI:** 10.3389/fnhum.2024.1418647

**Published:** 2024-07-16

**Authors:** Marco Muccio, Giuseppina Pilloni, Lillian Walton Masters, Peidong He, Lauren Krupp, Abhishek Datta, Marom Bikson, Leigh Charvet, Yulin Ge

**Affiliations:** ^1^Department of Radiology, NYU Grossman School of Medicine, New York, NY, United States; ^2^Department of Neurology, NYU Grossman School of Medicine, New York, NY, United States; ^3^Research and Development, Soterix Medical, Inc., Woodbridge, NJ, United States; ^4^Department of Biomedical Engineering, City College of New York, New York, NY, United States

**Keywords:** transcranial direct current stimulation, multiple sclerosis, MRI, neuronal metabolism, cerebrovascular changes, tDCS-aCT paired treatment

## Abstract

**Introduction:**

Transcranial direct current stimulation (tDCS) is a non-invasive neuromodulation technique with simultaneous (during stimulation) and cumulative effects (after repeated sessions) on blood flow and neuronal metabolism. These effects remain mostly unclear especially in multiple sclerosis (MS). This work aims to elucidate brain metabolic and hemodynamic underpinnings of tDCS and its potential therapeutic impact in MS patients using quantitative tDCS-MRI.

**Methods:**

MS participants (n = 20; age = 45.4 ± 12.3 years, 7 males) underwent 3 T MRI scans before and after 20 daily sessions of dorsolateral prefrontal cortex (DLFPC) tDCS (2.0 mA, left anodal) paired with adaptive cognitive training (aCT). During both visits, imaging measurements of cerebral blood flow (CBF), cerebral venous blood oxygenation (Yv) and calculated cerebral metabolic rate of oxygen (CMRO_2_) were obtained at pre-tDCS, during-tDCS and post-tDCS.

**Results:**

At baseline, significant increase from pre- to during-tDCS was observed in CMRO_2_ (7.6%; *p* = 0.002), CBF (11.0%; *p* < 0.0001) and Yv (1.9%; *p* = 0.006). At follow up, we observed an increase in pre-tDCS CMRO_2_ (140.59 ± 13.83 μmol/100 g/min) compared to baseline pre-tDCS levels (128.30 ± 14.00 μmol/100 g/min; *p* = 0.006). Sustained elevations in CMRO_2_ and CBF into post-tDCS were also observed (tDCS lingering effects). Cumulative tDCS effects were observed in the form of sustained elevations in CMRO_2_ and CBF in pre-tDCS follow up, reaching the magnitudes measured at baseline during-tDCS.

**Discussion:**

TDCS induces an acute surge in metabolic activity persisting immediately after the stimulation is removed. Moreover, treatment composed of repeated tDCS-aCT paired sessions contributes to establishing long-lasting increases in neuronal activity.

## 1 Introduction

Transcranial direct current stimulation (tDCS) is a safe and well-tolerated method of noninvasive brain stimulation where weak electrical currents are used to modulate cortical excitability (Nitsche et al., 2008; Bikson et al., 2016; Dedoncker et al., 2021). For behavioral or clinical effects, tDCS is often administered in repeated sessions to obtain cumulative effects over time (Ulam et al., 2015; Im et al., 2019), often paired with adaptive cognitive training (aCT) (Agarwal et al., 2018; Charvet et al., 2018; Eilam-Stock et al., 2021; Simani et al., 2022). There has been a growing clinical interest in its application for managing various neurological and psychological conditions (Fregni et al., 2021). However, as our understanding of the clinical applications of tDCS continues to progress, there is a need for further investigation into the corresponding biophysiological foundations.

New imaging techniques have been developed to calculate the global neuronal activity, namely cerebral metabolic rate of oxygen (CMRO_2_), from measurements of cerebral blood flow (CBF) and cerebral venous oxygenation (Yv). CMRO_2_ has been shown to be a reliable, quick method to accurately and non-invasively obtain measurements of brain metabolism levels (or neuronal activity) (Lu and Ge, 2008; Xu et al., 2009; Muccio et al., 2022). Such imaging advancements have facilitated the concurrent use of tDCS and MRI scanning, allowing for the assessment of cerebrovascular and neuronal characteristics during the stimulation (simultaneous effects). Early investigations using concurrent tDCS-MRI, have indeed reported tDCS-induced increases in regional and global CBF (Jamil et al., 2020) along with heightened neuronal activity in healthy controls (HC) (Muccio et al., 2022), also persisting beyond the cessation of stimulation (Nitsche and Paulus, 2001). Furthermore, recent neuroimaging studies have shown that repeated tDCS sessions lead to cumulative effects surpassing those observed following a single session. This has been reported in both healthy subjects (Alonzo et al., 2012) and clinical disorders with variability in treatment durations (Ulam et al., 2015; Im et al., 2019).

In the present study, we applied MRI methods to quantitatively assess the neuronal underpinnings of tDCS in patients with multiple sclerosis (MS). MS is a chronic and progressive condition that exhibits high variability in its onset and presentation, but typically begins in early adulthood (Tullman, 2013; Ford, 2020). TDCS shows particular promise in managing the symptom burden of MS, reducing the common symptom of fatigue (Charvet et al., 2018) and, when paired with rehabilitation training, improving both motor function (Pilloni et al., 2020) and cognitive performance (Simani et al., 2022). Few potential mechanisms could underlie tDCS-induced neuronal recovery in MS. In particular, this neurodegenerative disease is characterized by a gradual process of neuronal degeneration via progressive loss of myelin sheets (Bramow et al., 2010). Therefore, the diseased neurons might not be completely lost even if they cannot reach the action potential needed to trigger neuronal firing. TDCS could then provide the extra electrical stimulation needed to achieve firing potentials and “rehabilitate” these neurons into healthy activity, especially after repeated sessions. Previous studies have addressed this question (Nitsche et al., 2003; Bikson et al., 2019), however, further investigation is warranted to better understand the tDCS-linked neuronal recovery processes in MS and to compare such mechanisms with the effects of tDCS in HC.

Here we performed concurrent tDCS-MRI to investigate the neuronal and hemodynamic changes (CMRO_2_ and CBF) induced during the tDCS session (simultaneous effects) and the respective changes resulting from a repeated treatment (cumulative effects). Such treatment consisted of 20 daily sessions of tDCS targeting the left dorsolateral prefrontal cortex (DLPFC), combined with adaptive cognitive training (aCT) over the course of 1 month (Charvet et al., 2018).

We hypothesized that, at baseline, in participants with MS, tDCS would induce an increase in both CBF and CMRO_2_, with these increased levels persisting immediately after the stimulation (post-tDCS). However, we anticipated that these simultaneous effects may be attenuated at the follow up visit due to the expected cumulative effects of the treatment on CBF and CMRO_2_. We propose that investigating both simultaneous and cumulative tDCS effects using quantitative imaging measures of oxygen metabolism represents a robust approach for comprehensively assessing its impact on neuronal cells. This is crucial to inform its clinical use and guide its optimization as a therapeutic tool.

## 2 Methods

### 2.1 Participants

Potential participants with MS were recruited from the NYU Langone Health MS Comprehensive Care Center, National MS Society, and other local community referrals. Interested participants first completed a phone screening process, confirming MS diagnosis (any subtype) and score based on the expanded disability status scale (EDSS).

For this prospective observational study, imaging data was acquired from 20 patients with a diagnosis of MS (age = 45.4 ± 12.3 years, 7 males) at the NYU Langone Health Center for Biomedical Imaging, department of radiology. IRB approval was provided by NYU Langone Health and all the participants were recruited as part of a larger clinical study (study identifier: S18-005548). All participants underwent strict and specific recruiting criteria excluding patients with history of head injury, other neurological diseases, non-MRI-safe implants, or contraindications to tDCS. Such tDCS-linked contraindications are: skin disorder/sensitive skin (e.g., eczema, severe rashes), or other skin defects compromising the integrity of the skin at or near stimulation locations; treatment for a communicable skin disorder currently or over the past 12 months; pregnant or breastfeeding; current uncontrolled seizure disorders.

### 2.2 Experimental design and data acquisition

**Figure 1:**
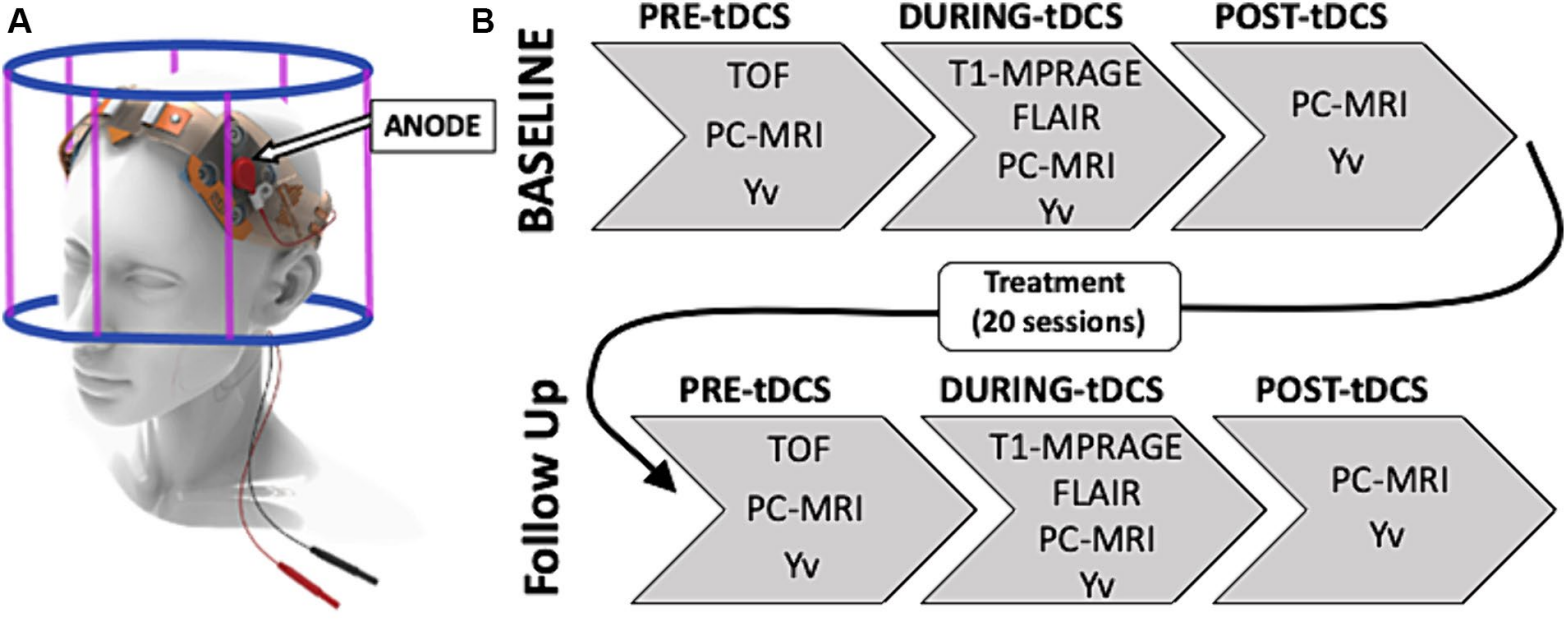
**(A)** TDCS montage with anode on the left dorsolateral prefrontal cortex (DLPFC; F3). **(B)** Study experimental design composed of a baseline concurrent tDCS-MRI session followed by a 20 at-home tDCS-paired treatment sessions and a follow up tDCS-MRI scan. During each MRI visit the image acquisition was divided in three sessions: before the stimulation (pre-tDCS), during 15 min 2.0 mA stimulation (during-tDCS) and right after turning off the stimulation (post-tDCS).

An MRI-compatible tDCS device (1×1 tDCS Model 1300A Low- Intensity Stimulator, Soterix Medical Inc.) was used to deliver the stimulation. This was done by applying two conductive rubber electrodes (5x5cm each), soaked in saline to reduce resistivity, over the forehead of the participant with anode aligned over the left dorsolateral prefrontal cortex (DLPFC, F3) and cathode over the right DLPFC (F4; Figure 1A). The current intensity chosen for the stimulation was 2.0 mA, commonly used in previous studies and shown to successfully influence neuronal activity (Kuo et al., 2020; Muccio et al., 2022). This was manually ramped up/down ensuring adequate contact quality (minimum impedance 8/10) between the electrodes and the skin at all times.

To acquire imaging data, we used a 3 T MRI (Prisma, Siemens) with a 64-channels head coil. Participants were asked to partake in a baseline 60 min concurrent tDCS-MRI session and then come back for a follow up visit. Between the two visits, participants received 20 at-home, remotely supervised tDCS sessions paired with adaptive cognitive training (aCT; BrainHQ). Stimulation duration and current intensity for these sessions were identical to the ones applied during the MRI scanning sessions.

Each tDCS-MRI visit was divided in a ‘pre-tDCS’ phase (15–20 min), during which no stimulation was given to the participant and a time of flight (TOF) was acquired to function as MR angiographic reference for the placement of the phase-contrast (PC) MRI imaging slice. After all imaging measures for pre-tDCS were successfully acquired, tDCS was manually ramped up, over a few seconds (<30s), to reach the current intensity of 2.0 mA before the start of the next scanning phase. In this second phase, ‘during-tDCS’ (15 min), the 2.0 mA tDCS was given to the participant and identical MRI sequences were used, with the addition of a 3D-T1 magnetization-prepared rapid acquisition gradient echo (MPRAGE; acquisition time-TA = 4min19sec, repetition time-TR = 2,300 ms, echo time-TE = 2.98 ms, flip angle = 9degrees, spatial resolution = 1x1x1mm) and 2D oblique axial fluid attenuated inversion recovery (FLAIR; acquisition time-TA = 2min44sec, repetition time-TR = 9,000 ms, echo time-TE = 2.98 ms, flip angle = 150degrees, spatial resolution = 0.7×0.7x2mm). This anatomical routine sequences were executed at the start of this phase to provide enough time for the stimulation to penetrate into the brain tissue. This approach was designed to ensure more reliable measures of tDCS-related dynamics for group-level comparisons by allowing the stimulation to reach a stable state in all patients at the moment of acquiring biophysiological imaging measures. This strategy mitigates variability among subjects in response to tDCS, particularly present in the initial moments after the stimulation begins. Finally, once all during-tDCS imaging sequences were completed, tDCS was manually ramped down (<30s) and turned off before proceeding with the ‘post-tDCS’ acquisition phase (15–20 min) which concluded the scanning session. In each phase, and each MRI visit, the same MRI sequences with identical scanning parameters were applied to allow adequate comparison of measurements. This study design is represented in Figure 1B.

**Figure 2:**
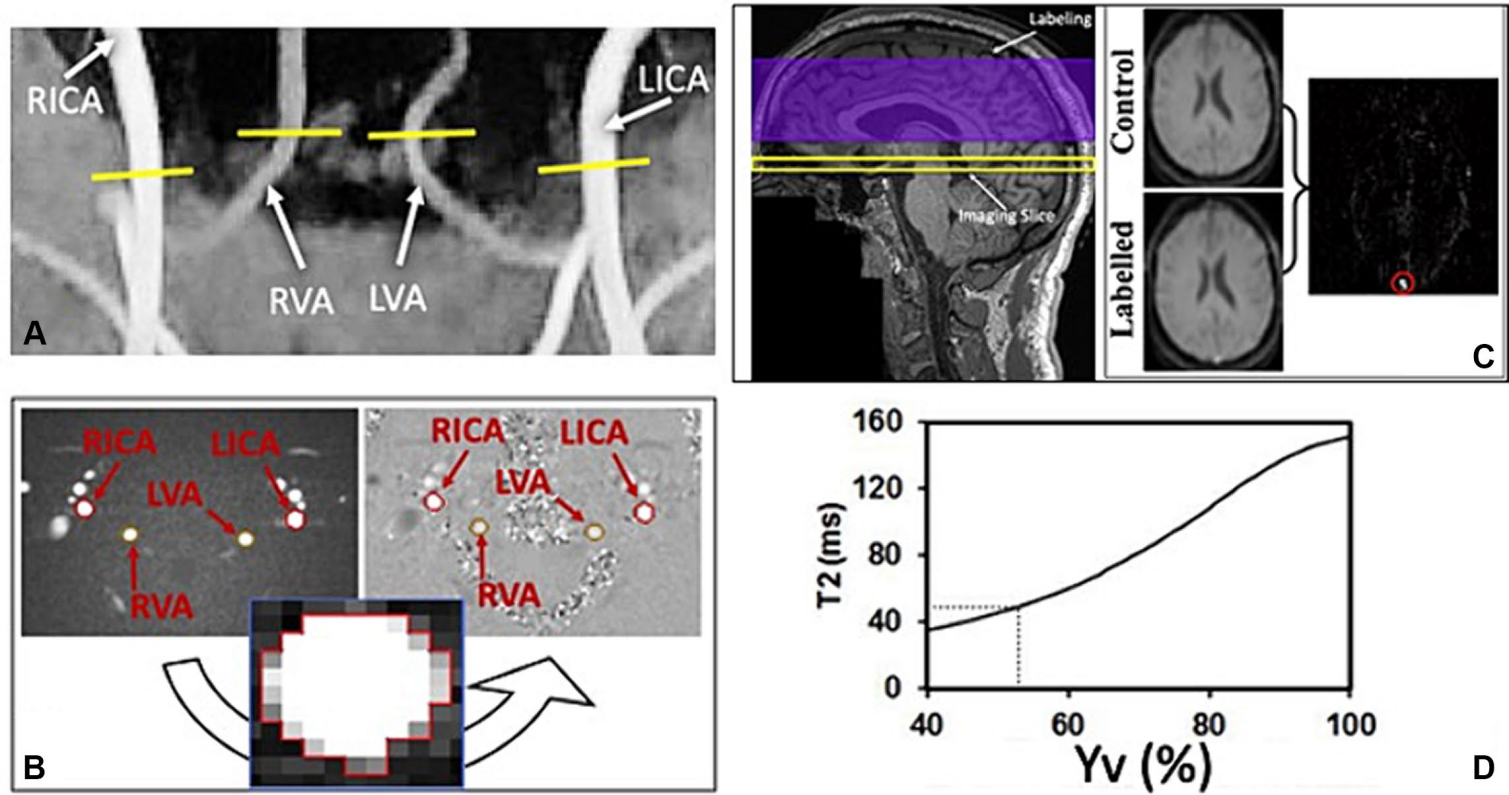
**(A)** Time of flight (TOF) coronal image shows four main neck arteries that are used to place the imaging slice of the phase contrast (PC) sequence. **(B)** Axial magnitude and phase images of PC-MRI used to quantify the blood flow of the 4 brain feeding arteries’ cross-sections: left and right internal carotid arteries (LICA and RICA); left and right vertebral arteries (LVA and RVA). **(C)** Imaging set up for the T2-Relaxation-Under-Spin- Tagging (TRUST) sequence on a representative structural (T1) image (left) and the obtained axial labelled and control magnitude images through the inferior superior sagittal sinus (SSS; right), used to obtain a linear subtraction image (labelled-control). This was then used to draw and ROI (red circle) around the SSS to extract the corresponding T2 values of the venous blood. **(D)** Calibration correlation curve of T2 signal used to estimate corresponding venous blood oxygenation (Yv).

Arterial blood flow was imaged using an axial PC-MRI sequence (TA = 10 s, TR = 25 ms, TE = 8 ms, slice thickness = 5 mm, flip angle = 15°, spatial resolution = 0.5×0.5x5mm; velocity encoding-VENC = 60 cm/s) encoding the velocity of the flowing spins on the blood flow within the main neck arteries, above the carotid bifurcation: bilateral carotid arteries (LICA and RICA) and bilateral vertebral arteries (LVA and RVA; Figure 2A).

T2-relaxation-under-spin-tagging (TRUST) (Lu and Ge, 2008) MRI was performed in a transverse plane parallel to the anterio-posterior commissure line, passing through the lower superior sagittal sinus (just above the confluence of sinuses) for a global estimation of venous oxygenation. Its imaging parameters were: TA = 87 s, TR/TE/TI = 3,000/19/1,200 milliseconds, FOV = 230 mm × 230 mm, matrix = 64 × 64, single-shot echo planar imaging, slice thickness = 5 mm, four enhanced echo times (eTEs): 0, 40, 80, and 160 milliseconds, corresponding to 0, 4, 8, and 16 refocusing pulses with an interval (τCPMG) of 10 milliseconds in the T2- preparation. Both sequences are described in more details in previous literature (Golay et al., 2001; Zhao et al., 2020).

### 2.3 MRI data analysis

All data were processed offline using in-house-written MATLAB (Mathworks, Natick, MA, United States) scripts. Structural images were analyzed using SPM12 (Wellcome Trust Center for Neuroimaging, Institute of Neurology, London United Kingdom) to extract gray matter (GM), white matter (WM) and cerebrospinal fluid (CSF) volumes (mL) needed to calculate the subject-specific brain parenchymal volume (BPV). Each subject’s lesion load was also extracted using the FireVoxel software (v314A, https://www.firevoxel.org/) unto which the 2D-FLAIR images were uploaded, and regions of interest (ROIs) were manually drawn by an expert radiologist (YG). PC-MRI images were used to quantify each artery’s blood flow. This measure was extracted by manually drawing a region of interest (ROI) around each individual artery’s cross-section, on the axial magnitude PC image. The ROI was then transposed to the corresponding phase image component and the average pixel intensity was used to calculate the average velocity of blood within the specific vessel (Figure 2B). For group comparisons, the extracted blood flow values were normalized using the individual specific BPV using Eq. 1, therefore obtaining a measurement of CBF.


(1) $$CBF=\frac{BF}{\rho \times \mathrm{BPV}\;}\times 100\;$$

Where CBF is the normalized blood flow; BF is the total blood flow output from the PC-MRI sequence; 𝜌 is the brain tissue density (1.06 g/mL) and BPV is the brain parenchymal volume obtained from 3D-T1-MPARAGE segmentation.

The details of data processing procedures for TRUST MRI and estimation of CMRO_2_ were described previously (Lu and Ge, 2008; Xu et al., 2009; Muccio et al., 2022). In short, the TRUST data consist of labeled and control images acquired at the lower part of the superior sagittal sinus, just above the confluence with the other sinuses. Each image type is acquired with four different eTEs. The signals from different eTEs were fitted to obtain a Carr-Purcell-Meiboom-Gill (CPMG) T2 of the venous blood (Figure 2C). Based on a well-established relationship between blood T2-relaxation time and blood oxygen saturation (Golay et al., 2001; Zhao et al., 2020), the measured venous blood T2 is then converted to the Yv using the calibration correlation shown in Figure 2D.

Using the data obtained for Yv and CBF, the index CMRO_2_ was then calculated. In this study, CMRO_2_ was calculated using Eq. 2, as previously reported by Xu *and Ge.* (2009) (Xu et al., 2009).


(2) $$CMRO_{2}=CBF\times \left(Y_{a}-Y_{v}\right)\times C_{a}$$

Where CBF is the normalized cerebral blood flow measured in mL/100 g/min; Ya and Yv are the arterial and venous blood oxygenation in % and Ca is a constant representing the amount of oxygen-carrying molecules per unit volume of blood. In males Ca = 8.562273 and in females Ca = 8.154545.

### 2.4 Statistical analysis

A Shapiro–Wilk test was initially carried out to assess the distribution of the measurements. A Wilcoxon matched-pairs rank test was used in comparisons involving measurements with non-normal distribution: CBF pre-(W = 0.83, *p* = 0.002) and during-tDCS (W = 0.79, *p* = 0.07) at follow up visit. For the other comparisons, a paired *t*-test was used to assess the hypothesized differences of these physiological factors (CBF, Yv and CMRO_2_) in pre- *vs* during-tDCS (simultaneous effects) and during- *vs* post-tDCS (lingering effects) at baseline and follow up separately as well as changes from baseline to follow up (cumulative effects). An independent t-test was used to investigate sex- related effects on tDCS modulation, comparing male and female participants’ data, and a Pearson’s linear correlation analysis was utilized to address the potential effects of age, brain volumes and lesion load on the measurements. Level of statistical significance was set at 0.05. Analysis and graphical representation were carried out using Matlab 2022a (The Mathworks Inc., Natick, Massachusetts United States) and GraphPad Prism version 9.0.0 for Mac (GraphPad Software, San Diego, California United States).

## 3 Results

**Figure 3:**
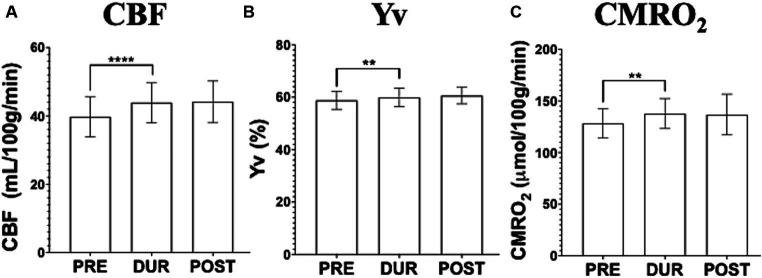
Imaging data acquired before (pre-tDCS), during (dur-tDCS) and after (post-tDCS) the stimulation to assess cerebral blood flow (CBF; **A**), venous blood oxygenation (Yv; **B**) and calculated cerebral metabolic rate of oxygen (CMRO_2_; **C**), at baseline. Notably, simultaneous tDCS effects, characterized by an increase from pre-tDCS to dur-tDCS, are observed across all three measures. Moreover, no difference was observed between during- and post-tDCS, suggesting lingering tDCS effects [^**^p < 0.01, ^****^p < 0.0001].

At baseline, we observed a significant increase in the global CBF of the MS participants from pre-(38.80 ± 5.75 mL/100 g/min) to during-tDCS (43.92 ± 5.74 mL/100 g/min; *p* < 0.0001). On the other hand, no significant change was observed in the post-tDCS CBF levels (44.17 ± 5.90 mL/100 g/min) compared to levels measured during the stimulation (*p* > 0.05; Figure 3A). For the measured venous oxygenation, or Yv, a slight increase was also observed from pre- (58.83 ± 3.39%) to during-tDCS (59.93 ± 3.34%; *p* = 0.006) and no changes in post-tDCS (60.59 ± 3.06%; *p* > 0.05; Figure 3B). Just as observed in CBF, pre-tDCS CMRO_2_ (128.30 ± 14.00 μmol/100 g/min) increased in during-tDCS (137.77 ± 14.17 μmol/100 g/min; *p* = 0.002) and remained unchanged in post-tDCS (136.80 ± 19.25 μmol/100 g/min; *p* > 0.05; Figure 3C).

**Figure 4:**
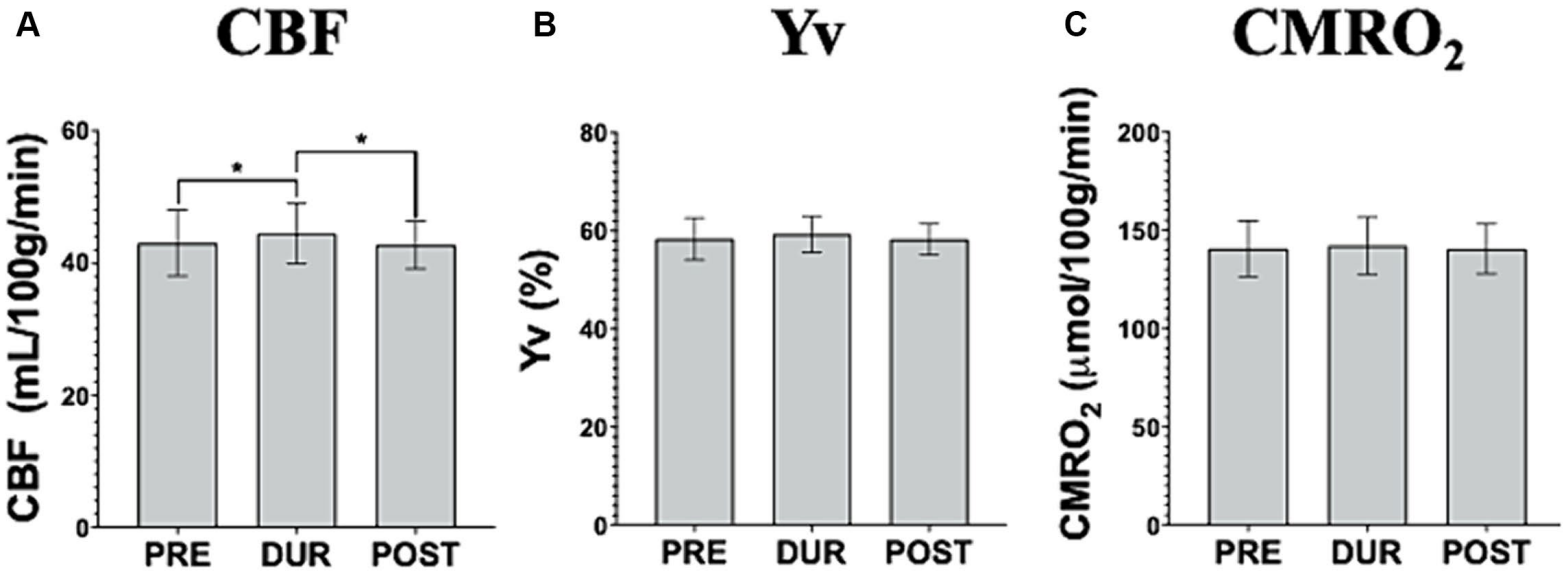
Imaging data acquired pre-, dur- and post-tDCS to assess cerebral blood flow (CBF; **A**), venous blood oxygenation (Yv; **B**) and calculated cerebral metabolic rate of oxygen (CMRO_2_; **C**), during the follow up visit. It is noteworthy that no simultaneous tDCS effects are observed in CMRO_2_ or Yv. Interestingly, CBF demonstrates an increase during the stimulation and a subsequent decrease upon tDCS cessation, back to pre-tDCS levels [^*^p < 0.05].

At follow up, after undergoing repeated tDCS-aCT sessions, different tDCS simultaneous effects were observed. Here, a small but significant increase in CBF was observed in during-(44.44 ± 4.41 mL/100 g/min) compared to pre-tDCS (43.02 ± 4.85 mL/100 g/min; *p* = 0.014) followed by another small but significant decrease in post- (42.70 ± 3.52 mL/100 g/min) compared to during-tDCS (*p* = 0.044; Figure 4A). However, no statistically significant changes were observed throughout the follow up scanning session in neither Yv (pre-tDCS = 58.34 ± 4.18%; during-tDCS = 59.31 ± 3.63%; post-tDCS = 58.27 ± 3.07%; *p* > 0.05; Figure 4B) nor CMRO_2_ (pre-tDCS = 140.59 ± 13.83 μmol/100 g/min; during-tDCS = 142.02 ± 14.02 μmol/100 g/min; post-tDCS = 140.44 ± 12.33 μmol/100 g/min; *p* > 0.05; Figure 4C).

**Figure 5:**
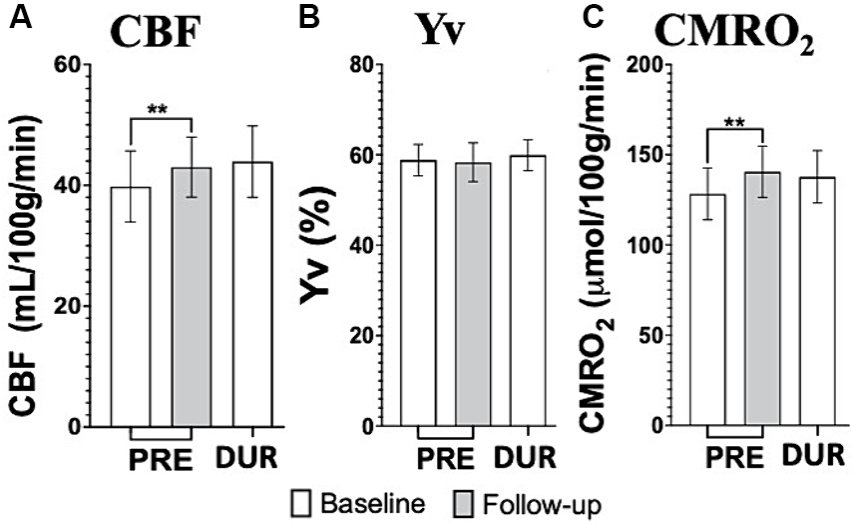
tDCS imaging results from cerebral blood flow (CBF; **A**), venous blood oxygenation (Yv; **B**) and calculated cerebral metabolic rate of oxygen (CMRO_2_; **C**) at baseline (white bars) and follow up (gray bars). Notably, the repeated application of tDCS, through daily sessions, significantly elevates both CBF and CMRO_2_ levels of pre-tDCS at follow up to magnitudes not different than what measured during-tDCS at baseline. This provides substantial support for the hypothesized cumulative effects induced by tDCS [^**^p < 0.01].

Cumulative effects showed significantly increased CBF and CMRO_2_ pre-tDCS levels, (*p* = 0.0098 and p = 0.006 respectively), before re-applying the stimulation, from baseline to follow up, which was not observed in Yv (Figures 5A–C). Notably, no significant differences were observed between follow up pre-tDCS and baseline during-tDCS in CMRO_2_ (*p* > 0.59) nor CBF (*p* > 0.58).

No other noteworthy significant differences were observed in cumulative effects for these three parameters (*p* > 0.05). Imaging measurements for both visits (baseline and follow up) are summarized in Table 1, with respective *p*-values from the paired *t*-test comparing pre- *vs* during-tDCS (simultaneous effects) and baseline *vs* follow up (cumulative effects).

**Table 1:** Simultaneous and cumulative effects of tDCS and tDCS-aCT treatment.

		Pre (mean ± SD)	During (mean ± SD)	% Change (mean)	*p*-values (simultaneous)
CBF (mL/100 g/min)	Baseline	38.80 ± 5.75	43.92 ± 5.74	11.01	<0.0001
	Follow up	43.02 ± 4.85	44.44 ± 4.41	3.58	0.014
	*p*-values (*cumulative*)	0.0098	0.7012	0.017	N.A.
Yv (%)	Baseline	58.83 ± 3.39	59.93 ± 3.34	1.92	0.006
	Follow up	58.34 ± 4.18	59.31 ± 3.63	1.86	0.139
	*p*-values (*cumulative*)	0.675	0.530	0.967	N.A.
CMRO_2_ (μmol/100 g/min)	Baseline	128.30 ± 14.00	137.77 ± 14.17	7.56	0.002
	Follow up	140.59 ± 13.83	142.02 ± 14.02	1.16	0.424
	*p*-values (*cumulative*)	0.006	0.411	0.014	N.A.

Simultaneous refers to comparisons of pre and during tDCS measurements (CBF, Yv or CMRO_2_) for each MRI session individually (baseline or follow up) *Cumulative* refers to comparisons of baseline and follow up measurements at either Pre or during tDCS. Post-tDCS measurements are not reported to ensure table readability and since they did not significantly differ from during-tDCS measures (*p* > 0.05). Significance threshold for *p*-value set at 0.05 and N.A., non applicable.

Statistical analyses investigating the contribution of demographic (age and sex) and physiological factors (brain volumes and lesion load) were also carried out. Unpaired *t*-test comparing responses to tDCS in males *vs* females did not show any sex differences (both *p* > 0.05). Linear regression analysis did not show any noteworthy correlations between our measurements and parameters such as age, brain volumes and lesion load (all *p* > 0.05 and *R*^2^ < 0.3).

## 4 Discussion

Recently there has been a burgeoning interest in tDCS applications in several neurological and psychological diseases such as depression (Zhang et al., 2021), attention deficit hyperactivity disorder (ADHD) (Breitling et al., 2016), stroke (O'Shea et al., 2014), Parkinson’s Disease (PD) (Ishikuro et al., 2018) and MS. In the latter, preclinical studies have even reported instances of tDCS-assisted neuronal remyelination (Mojaverrostami et al., 2022). In this specific study, we used advanced imaging techniques to quantify the neuronal influences of left anodal DLPFC tDCS as result of the external stimulation itself (simultaneous effects) and of aCT paired treatment via repeated sessions (cumulative effects), in MS patients.

### 4.1 Simultaneous effects

In the first tDCS-MRI visit (baseline) we observed an immediate increase in CBF during the 15 min 2.0 mA tDCS application. Although similar increase was observed in Yv, likely as consequence of the extra oxygen supplied by the increased CBF, such change was not proportionate to the blood flow change (11.01% CBF *vs* 1.92% Yv). It follows that most of the surplus oxygen was readily utilized by the stimulated neurons, leading to the observed increase in CMRO_2_ (7.56%) indexing for neuronal activity. This supports the current understanding that anodal tDCS causes an acute increase in neuronal firing by further depolarizing the neuronal membrane (Nitsche and Paulus, 2000; Jamil and Nitsche, 2017) and is in line with other studies investigating the functional and structural connectivity changes due to tDCS (Sankarasubramanian et al., 2017; Hirtz et al., 2018; Kim et al., 2021; Jog et al., 2023).

Notably, a previous study using the same concurrent tDCS-MRI paradigm on healthy controls (HCs) (Muccio et al., 2022) also reported a global CMRO_2_ increase during the stimulation. However, these tDCS-induced neuronal change (5.9%) is considerably smaller than that observed in MS patients in this study. Such difference in response to tDCS between MS and HCs can be seen as a representation of the hypothesized larger potential for neuronal plasticity of MS patients. We posit this hypothesis based on the expectation that healthy neurons operate at already optimal capacity, whereas the demyelinated neurons of MS patients, which might have not yet completely lost their functions, may require external stimulation to achieve comparable firing levels. However, more systematic and direct studies are necessary in order to test the hypothesis mentioned here of a larger increase in neuronal activity in MS compared to HCs during tDCS.

At the follow up visit, no simultaneous tDCS effects were significant on the neuronal activity of the MS participants. CBF analysis, on the other hand, showed a small but significant increase during tDCS, followed by another small but significant decrease in post-tDCS. This particular observation suggests that tDCS might have different effects on the cerebrovascular properties which might be independent of the more direct neuronal ones, as recently suggested (Merzagora et al., 2010). This consequently warrants further studies to disentangle the cerebrovascular and neuronal effects of tDCS.

Our study also offers strong support for the persistence of tDCS’ neuronal effects even after the stimulation is removed, known as lingering effects. Specifically, the increase in CMRO_2_ measured during tDCS remained elevated in the post-tDCS phases in both visits, consistent with previous literature reports (Zappasodi et al., 2019; Chan et al., 2021). However, it’s important to note that these lingering effects are known to be influenced by specific stimulation parameters, such as dosage (Jamil et al., 2017). We hypothesize that these effects may further elucidate the cumulative impact of repeated tDCS sessions and contribute to distinguishing responses to tDCS treatment even when paired to cognitive or motor training. Further studies are required to explore this hypothesis.

### 4.2 Cumulative treatment-induced effects

**Figure 6:**
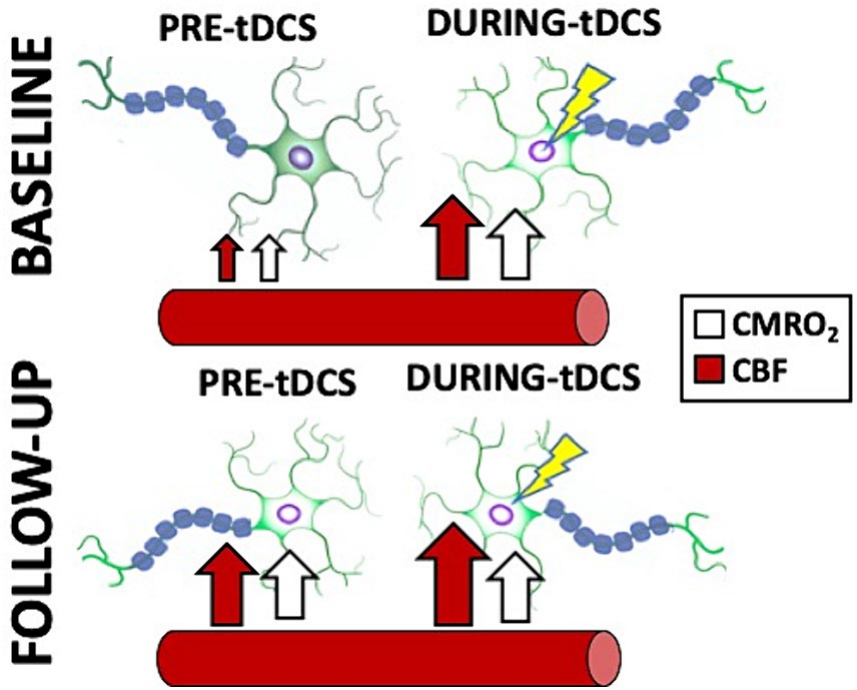
Cartoon representing a summary of the simultaneous and cumulative effects of tDCS as reported in this study. Notice how the stimulated neurons immediately increase their firing in response to tDCS (CMRO_2_ increase; white arrows), facilitated by an increase in blood supply to the brain (CBF increase; red arrows). Additionally, note how the treatment, involving repeated tDCS sessions, causes the neurons to remain in the stimulated state with increased activity measured pre-tDCS, even at follow up. Interestingly, the re-introduction of the stimulation does not result in a further increase in CMRO_2_, but does induce a slight increase in CBF.

MS participants exhibited evident signs of the treatment-related cumulative effects measured with our innovative imaging techniques. In this study, tDCS-aCT treatment contributed to an increase of pre-tDCS levels at follow up for both CBF (10.9%) and CMRO_2_ (9.6%) compared to pre-tDCS at baseline. With the imaging techniques in this study, cumulative effects of tDCS-paired treatment were observed to contribute to establishing a long-lasting elevation of the patients’ neuronal activity, possibly by extension of the lingering effects from each of the 20 sessions. Such cumulative changes also explain why we did not observe any neuronal simultaneous changes at follow up. Notably, we observed no statistically significant difference (*p* > 0.05) between the CMRO2 measures at baseline during-tDCS and follow up pre-tDCS. This might imply that the effectiveness of repeated tDCS sessions might cause cumulative, long-lasting effects on neuronal activation mirroring the simultaneous effects observed at baseline. In addition, we suggest that the overall neuronal effects of tDCS can be limited by the stimulation specific parameters which cannot be exceeded by their accumulation, consequently implying the existence of a possible plateau of tDCS effectiveness. The upper limit of such effects might then be given to the particular tDCS parameters chosen (e.g., dosage, montage, directionality) and this is further supported by the fact that the increased pre-tDCS level measured at follow up is considerably similar to what measured in during-tDCS at baseline. Figure 6 provides comprehensive representation of the simultaneous and cumulative effects of tDCS, encompassing both neuronal and cerebrovascular aspects. Additionally, we did not find the correlation between neuronal response to tDCS and lesion load, even though there is lesion-related difference of neuroplasticity potential among MS participants (Saiote et al., 2014). Further studies are necessary to address this question, as such correlations are rarely investigated and results are still controversial.

Despite our study’s relatively small sample size, which is comparable to or larger than that of previous studies, there are some other limitations that should be acknowledged. For instance, we cannot assert whether the imaging results obtained are specific to the chosen tDCS parameters, such as duration, intensity, or montage (Furubayashi et al., 2008; Ammann et al., 2017; Shinde et al., 2021). While the inclusion of a sham (control) arm could have enhanced reliability of our results, this study specifically aims to underscore the importance of imaging applications in understanding the mechanistic aspect of tDCS, focusing on within-subject changes. Moreover, it is still not well established whether sham-tDCS has no biophysiological effects resulting from the brief application of the stimulation and its reliability is still debated in the literature (Loo et al., 2018; Fonteneau et al., 2019; Turi et al., 2019; De Smet et al., 2021). Furthermore, it is recognized that other physiological factors, such as arterial structure and body position might affect the hydrodynamic properties of the brain besides tDCS (Muccio et al., 2021; Sun et al., 2022). These factors might play a significant role in our measurements. Additionally, the inclusion of cognitive training as part of the treatment does not allow us to conclusively discern the neuroimaging changes induced by tDCS alone. Previous studies have in fact shown increases in CBF as result of adaptive cognitive training alone (Mozolic et al., 2010). Nevertheless, it is known that such paired treatment (tDCS-aCT) leads to greater clinical and metabolic changes compared to cognitive training alone (Charvet et al., 2018; Nissim et al., 2019; Simani et al., 2022).

Further studies should expand on our findings and more directly address the dynamic after effects of tDCS at multiple timepoints immediately after the start and end of the stimulation phase; potential correlations between response to tDCS-treatment and higher current intensities; as well as disentangling the cerebrovascular and strictly neuronal properties of the stimulation. Studies with larger sample sized might also investigate whether imaging measures could be used to differentiate tDCS responders and non-responders.

## 5 Conclusion

In conclusion, we have demonstrated that tDCS induces acute neuronal response in MS patients and that such effects accumulated over time as result of repeated tDCS paired with adaptive cognitive training. This can be successfully and efficiently captured using advanced imaging techniques (e.g., CMRO_2_). Furthermore, this study establishes a foundation for a more in-depth exploration into whether the concurrent impact of tDCS can serve as a predictor for its cumulative effects. Our view lies in the potential of using such advanced imaging techniques to classify subjects undergoing tDCS treatment as respondent or non-respondents, leveraging their initial (baseline) response to stimulation. This, in turn, holds promise for offering insights that could guide future clinical treatment decisions.

## Data availability statement

The datasets presented in this article are not readily available because of identifiable and highly confidential nature of the imaging dataset. Data has been stored in secured and encrypted servers. Requests to access the datasets should be directed to YG, Yulin.Ge@nyulangone.org.

## Ethics statement

The studies involving humans were approved by NYU Langone Health (study identifier: S18-005548). The studies were conducted in accordance with the local legislation and institutional requirements. The participants provided their written informed consent to participate in this study.

## Author contributions

MM: Data curation, Formal analysis, Investigation, Methodology, Validation, Visualization, Writing – original draft, Writing – review & editing. GP: Investigation, Project administration, Writing – review & editing. LW: Data curation, Investigation, Project administration, Writing – review & editing. PH: Data curation, Investigation, Writing – review & editing. LK: Writing – review & editing. AD: Writing – review & editing. MB: Writing – review & editing. LC: Conceptualization, Funding acquisition, Resources, Supervision, Validation, Writing – review & editing. YG: Conceptualization, Funding acquisition, Investigation, Methodology, Supervision, Validation, Visualization, Writing – original draft, Writing – review & editing.
